# Is infant exposure to antiretroviral drugs during breastfeeding quantitatively important? A systematic review and meta-analysis of pharmacokinetic studies

**DOI:** 10.1093/jac/dkv080

**Published:** 2015-04-08

**Authors:** Catriona John Waitt, Paul Garner, Laura Jayne Bonnett, Saye Hock Khoo, Laura Jayne Else

**Affiliations:** 1 Department of Molecular and Clinical Pharmacology, University of Liverpool, Block A, The Waterhouse Buildings, 1–5 Brownlow Street, Liverpool L69 3GE, UK; 2 Clinical Sciences Department, Liverpool School of Tropical Medicine, Pembroke Place, Liverpool L3 5QA, UK; 3 Department of Biostatistics, University of Liverpool, Faculty of Health and Life Sciences, 1st Floor Duncan Building, Daulby Street, Liverpool L69 3GA, UK; 4 Department of Clinical Infection, Microbiology and Immunology, University of Liverpool, 8 West Derby Street, Liverpool L69 7BE, UK

**Keywords:** ARV, mother-to-child transmission, PK

## Abstract

**Objectives:**

The objectives of this study were to summarize antiretroviral drug concentrations in breast milk (BM) and exposure of breast-fed infants.

**Methods:**

This was a systematic review of pharmacokinetic studies of HIV-positive women taking antiretrovirals that measured drugs in BM. The quality of pharmacokinetic and laboratory methods was assessed using pre-defined criteria. Pooled ratios and 95% CIs were calculated using the generalized inverse variance method and heterogeneity was estimated by the *I*^2^ statistic. PubMed Central, SCOPUS and LactMed databases were searched. No date or language restrictions were applied. Searches were conducted up to 10 November 2014. Clinical relevance was estimated by comparing ingested dose with the recommended therapeutic dose for each drug.

**Results:**

Twenty-four studies were included. There was substantial variability in the clinical and laboratory methods used and in reported results. Relative to maternal plasma (MP), NRTIs accumulate in BM, with BM : MP ratios (95% CI estimates) from 0.89 to 1.21 (14 studies, 1159 paired BM and MP samples). NNRTI estimates were from 0.71 to 0.94 (17 studies, 965 paired samples) and PI estimates were from 0.17 to 0.21 (8 studies, 477 paired samples). Relative to the recommended paediatric doses, a breast-fed infant may ingest 8.4% (95% CI 1.9–15.0), 12.5% (95% CI 2.6–22.3) and 1.1% (95% CI 0–3.6) of lamivudine, nevirapine and efavirenz, respectively, via BM.

**Conclusions:**

Transfer to untreated infants appears quantitatively important for some NRTIs and NNRTIs. The pharmacokinetic methods varied widely and we propose standards for the design, analysis and reporting of future pharmacokinetic studies of drug transfer during breastfeeding.

## Introduction

Approximately 1.5 million HIV-positive women become pregnant each year.^1^ The infection of some 400 000 infants annually led the WHO to encourage provision of efavirenz-based antiretroviral (ARV) therapy for pregnant HIV-positive women [prevention of mother-to-child transmission (PMTCT) options B and B+].^2^ Increasing numbers of women will receive ARVs throughout breastfeeding.

In contrast to well-resourced settings,^3–5^ the WHO recommends exclusive breastfeeding in the developing world (since formula feeding is associated with high infant mortality)^6^ despite breastfeeding accounting for a significant proportion of all mother-to-child transmissions of HIV.^5^ Infants of ARV recipients who acquire HIV via breast milk (BM) have high rates of drug resistance,^7,8^ limiting treatment options and shortening life. Pharmacokinetic (PK) knowledge of ARV transfer to BM and breast-fed infants is essential to understand the safety of prolonged exposure through breastfeeding and limit the development of drug resistance.

The objectives of this study were to summarize from existing studies of breastfeeding mothers taking ARVs: (i) ARV concentrations in BM; and (ii) ARV transfer to breast-fed infants.

## Methods

We wrote the protocol for this review before starting the analysis (available as Supplementary data at *JAC* Online), following PRISMA guidelines.^9^

### Ethics

Ethics approval was not necessary for this systematic review.

### Inclusion criteria

We sought to identify and analyse all studies that reported drug concentrations of any ARV in the BM of HIV-positive women on ARVs.

### Search strategy

PubMed Central, SCOPUS and LactMed databases were searched using the keywords ‘antiretroviral’ and ‘breast’ and ‘milk’ and subsequently by replacing the generic term ‘antiretroviral’ with the name of each individual agent. No date or language restrictions were applied. The proceedings of relevant conferences and citation lists from review articles and included papers were searched. Searches were conducted up to 10 November 2014.

### Data extraction

Data were extracted onto an Excel^®^ spreadsheet by C. J. W. and L. J. E., with differences resolved by discussion amongst all authors. For each study, the author, date, country, study design including inclusion and exclusion criteria, sample size, ARV regimen(s) administered to the mother and whether or not infant ARVs were administered were recorded.

### Methodological rigor and reporting quality

For clinical sampling, we assessed: (i) the PK sampling strategy in terms of number of BM samples obtained from each mother, timing of sampling relative to birth and to maternal dosing, or whether this was unclear; (ii) if the methods used to obtain BM were described sufficiently (volume of milk, manual or pump-assisted expression and storage of milk) for another investigator to be able to replicate the process; and (iii) whether the samples of maternal plasma (MP), infant plasma (IP) and BM were obtained at the same time, a different time or if this was unclear.

We assessed the quality of the laboratory methods using seven criteria: (i) if the laboratory method was described in sufficient detail for another investigator to be able to repeat the procedure; (ii) whether the authors validated or referred to assay validation for the BM matrix (yes/no); (iii) if drug was measured in whole BM, if the lipid fraction was skimmed off or if both of these methods were used; (iv) the method of drug extraction; (v) the choice of detection method used; (vi) if the assay sensitivity was reported (yes/no); and (vii) if detail regarding the internal standard was provided (yes/no).

### Data analysis

#### Outcomes

Medians and IQRs for ARV concentrations in human BM, MP and IP were extracted. The ratio of ARV in BM to MP (BM : MP ratio) is accepted as the key index of BM transfer of drug;^10^ where this was not specifically stated, it was calculated from median MP and BM concentrations where these were provided. BM : MP and IP : BM ratios were only determined when MP, BM and IP concentrations were detectable (above the assay limit of quantification). Hence not all studies provided sufficient data for inclusion in the statistical analysis or presentation in the figures.

#### Statistical analysis

The individual studies identified in this review provided a median and IQR or single point estimates for drug concentrations and BM : MP ratios. Therefore, to provide a pooled estimate for the BM : MP and IP : BM ratios, it was necessary to assume that the ratios were normally distributed and that the median is approximately equal to the mean. Additionally, it is assumed that the IQR is four-thirds of the standard deviation, based on the fact that the standard normal has its 25th and 75th percentiles approximately two-thirds of a standard deviation away from zero.^11^

Having calculated an assumed mean and standard deviation for all relevant studies, pooled estimates of each outcome were obtained via the generalized inverse variance method. Studies without a spread measure were removed from the pooling exercise.

To estimate clinical relevance, BM concentrations were interpreted as the percentage of the recommended infant dose^12^ that would be ingested by a fully breast-fed infant. If both treatment and prophylactic doses were available, we used the PMTCT dose reflecting that which a neonate might typically be prescribed. Whilst not included in WHO guidelines, the FDA has approved efavirenz use in infants aged >3 months and weighing >3.5 kg under exceptional circumstances. Genotyping for CYP2B6 metabolizer status is strongly recommended with appropriate dose adjustment, but if not available a dose of 100 mg would be used for a 3.5 kg infant;^13^ this was used in our calculations. The standard assumption of 150 mL/kg/day milk intake was made. As studies did not summarize infant weights, simulations were made for infants weighing 2, 3, 4 and 5 kg. CIs for the percentages were calculated using standard methodology and once again pooled estimates were obtained using the generalized inverse variance method with zero percentages being excluded from the pooling. CIs were capped at 0 and 100 as true percentages are being considered, not changes in percentages.

Heterogeneity was estimated via the *I*^2^ statistic. It was not possible to calculate an *I*^2^ statistic where there is only a single study or where only one of several studies for a drug has a measure of spread.

## Results

### Description of studies

**Figure 1.**
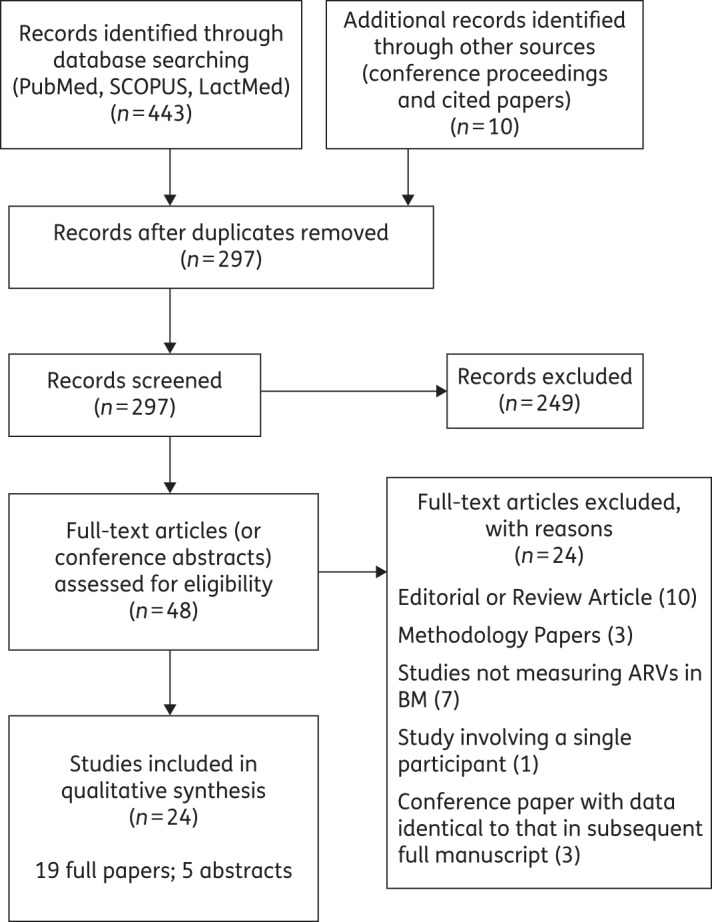
Information sources and search strategy.

Twenty-four studies (19 full text and 5 conference proceedings) met the inclusion criteria and are summarized in Table 1. Figure 1 illustrates the information sources and search strategy. Fourteen were PK studies nested within PMTCT efficacy trials, 8 were observational PK studies and 2 were nested within early-phase clinical trials. Nineteen studies were conducted in sub-Saharan Africa.

**Table 1.** Study population

Study population						Drugs taken by mother					
study	country	design	inclusion criteria	exclusion criteria	size	duration of ART	NVP	ZDV	3TC	other	Drugs taken by infant
Aizire^46^	Uganda	observational	ARV naive, CD4 >250 cells/mm^3^	multiple pregnancy; in a clinical trial; partner refusal	120 pairs	single perinatal dose	X				not stated
Benaboud^34^	Cote d'Ivoire	nested in RCT	not stated (within trial population)	not stated	5 mothers	7 days	X			FTC, TDF	not stated
Colebunders^18^	Belgium	observational	on HAART	not stated	10 mothers	until after BM sampling	X	X	X	NFV, IDV	ZDV single dose
Corbett^a22^	Malawi	nested in RCT	CD4 >200 cells/mm^3^; infant birth weight >2 kg	not stated	20 pairs	7 days or throughout breastfeeding (RCT)	X	X	X	d4T, NFV	NVP single dose + ZDV/3TC 7 days
Corbett^16^	Malawi	nested in RCT	CD4 >200 cells/mm^3^; on ARVs, infants HIV−	not stated	30 pairs	delivery to end of breastfeeding		X	X	LPV/r	not stated
Fogel^32^	Malawi	nested in RCT	CD4 <250 cells/mm^3^	not stated	52 pairs	median 1.5 months at sampling time				d4T	NVP (one of three regimens in PEPI-Malawi trial)
Frank^47^	Uganda	observational	ineligible for HAART	not stated	62 pairs	single perinatal dose	X				NVP 2 mg/kg
Giuliano^24^	Mozambique	observational	on any PMTCT regimen	not stated	40 women	28 weeks gestation–1 month post-partum	X	X	X		NVP 2 mg/kg
Kunz^20^	Uganda	nested in RCT	not stated (trial population)	on HAART	62 pairs	single perinatal dose	X				NVP 2 mg/kg
Mirochnick^48^	USA and Puerto Rico	Phase I	ARV naive; enrolled at >34 weeks gestation	intercurrent illness; significant fetal anomaly; lab abnormalities	3 pairs	single perinatal dose	X				NVP 2 mg/kg
Mirochnick^25^	Kenya	nested in RCT	not stated (trial population)	not stated	67 pairs	from ∼34 weeks gestation through 6 months breastfeeding	X	X	X		NVP 2 mg/kg
Mirochnick^49^	Malawi + Brazil	nested in RCT	not stated (trial population)	previous TDF; condition that might affect PK	25 mothers	single perinatal dose				TDF	TDF 4 mg/kg on days 1, 3 and 5
Moodley^29^	South Africa	nested in RCT	ARV naive	multiple pregnancy; significant fetal anomaly; lab abnormalities	20 pairs	from ∼38 weeks gestation until 1 week post-partum		X	X		3TC 4 mg/kg bd alone or with ZDV 2 mg/kg qds for 1 week
Musoke^50^	Uganda	Phase I/II study	ARV naive; enrolled at >34 weeks gestation	intercurrent illness; significant fetal anomaly; lab abnormalities	21 pairs	single perinatal dose	X				NVP 2 mg/kg
Olagunju^a28^	Nigeria	observational	on EFV-based ARV; exclusively breastfeeding	not stated	51 pairs	not stated			X	EFV, FTC, TDF	not stated
Palombi^23^	Malawi	nested in RCT	not stated (trial population)	lab abnormalities	66 pairs	from 25 weeks gestation until end of breastfeeding	X	X	X	d4T, LPV/r	NVP 2 mg/kg
Rezk^31^	Malawi	nested in RCT	on HAART	not stated	60 women	not stated	X	X	X	d4T, NFV, LPV/r	not stated
Ruff^a17^	Haiti	observational	not stated	not stated	6 women	single dose	X				no
Schneider^27^	Rwanda	nested in RCT	>45 days since delivery	not stated	13 pairs	from ∼28 weeks gestation until 6 months post-partum	X	X	X	EFV	NVP 2 mg/kg stat + ZDV 4 mg/kg for 7 days
Shapiro^45^	Botswana	nested in RCT	not stated (trial population)	not stated	50 pairs	from 24–36 weeks gestation, throughout breastfeeding	X	X	X	ABC, LPV/r	NVP 6 mg, ZDV 4 mg/kg bd for 4 weeks
Shapiro^30^	Botswana	nested in RCT	on HAART	not stated	20 pairs	>6 weeks	X	X	X		ZDV 4–6 mg tds
Spencer^a15^	USA	observational	on HAART	not stated	7 women	not stated		X	X	ATV	not stated
Spencer^a14^	USA	observational	stable on HAART; undetectable viral load	not stated	9 women	post-partum days 1–14				ETR	not stated
Weidle^51^	Kenya	nested in RCT	not stated (trial population)	not stated	26 pairs	34 weeks gestation to 6 months post-partum				NFV	not stated

NVP, nevirapine; EFV, efavirenz; ETR, etravirine; ZDV, zidovudine; 3TC, lamivudine; d4T, stavudine; TDF, tenofovir; FTC, emtricitabine; ABC, abacavir; ATV, atazanavir; IDV, indinavir; LPV/r, ritonavir-boosted lopinavir; NFV, nelfinavir; RCT, randomized controlled trial; bd, twice daily dosing; tds, three times daily dosing; qds, four times daily dosing; stat, single dose.

^a^Conference proceeding.

The individual ARVs analysed in breastfeeding mothers were nevirapine (16 studies), zidovudine (12 studies), lamivudine (12 studies), stavudine (4 studies), lopinavir (4 studies), ritonavir (4 studies), nelfinavir (4 studies), tenofovir (2 studies), efavirenz (2 studies), atazanavir (1 study), indinavir (1 study), abacavir (1 study) and etravirine (1 study).

Eight studies reported IP concentrations where the infants were not given the same drug as the mother. A further seven also reported IP concentrations where infants and mothers were receiving the same drug; here, MP and BM concentrations were retained and IP levels excluded, as drug exposures in the infant would reflect both orally administered drug and BM transfer, making it impossible to determine the contribution from the mother via BM. In two studies, infant blood sampling was performed at intervals over a period of several months; in this case, we included in the analysis only timepoints where the directly administered infant drug would have no longer been detectable based on the known ARV elimination half-life.

### Study design

The PK study design, types of matrices analysed (MP, BM and IP) and the laboratory methods are described in Tables S1 and S2.

### PK design

For BM PK, 13 studies utilized a sparse PK sampling strategy (a single timepoint within a dosing interval), with sampling either within the first 6 weeks of life (6 studies) or at intervals up to 6 months of age (7 studies). Two studies undertook a rich PK profile on BM, sampling at 0, 2, 5, 8 and 24 h relative to dosing,^14,15^ and two further studies performed truncated rich PK analysis from 0 to 6 h post-dose.^16,17^ In seven studies, the PK design was not clear. Fifteen studies sampled all three matrices (MP, BM and IP), whereas 9 did not include infant concentrations. Twenty studies sampled the different matrices contemporaneously and 3 did not; in the final study, this was not clear (Table S1).

### Methods to obtain and process BM

Nine studies provided detail on the clinical process of BM sampling (Table S1). Regarding the fraction of milk analysed, three studies reported the measurement of drug in BM with the lipid layer skimmed off, six reported measurement in whole BM (one of which specified that this was homogenized prior to extraction)^18^ and three studies considered both whole and skimmed BM. In the remaining 12 studies, the milk fraction was not specified (Table S2).

### Quality of laboratory methods

We evaluated whether matrix-specific assay validation for BM was described, in accordance with FDA bioanalysis guidelines.^19^ The majority of methods were either LC-MS or HPLC with ultraviolet detection based; one study employed GC-MS. Two studies described the use of drug-free milk for use in assay optimization.^20,21^ Fourteen studies described assay validation or referenced validated methods. Fourteen studies specified the assay sensitivity in terms of the lower limit of quantification and eight studies specified the internal standard used (Table S2).

### Disposition of ARVs into BM and infant

**Figure 2.**
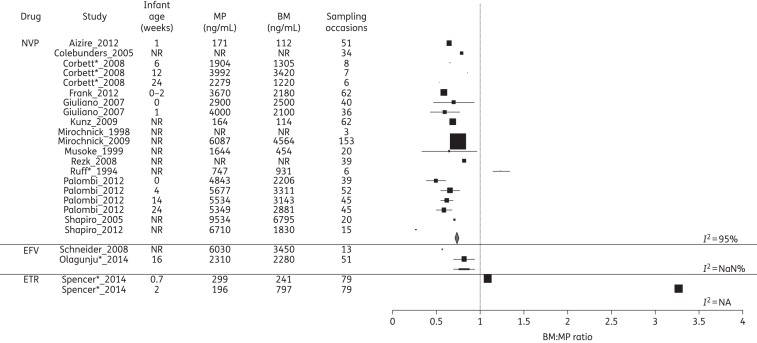
Forest plot of BM : MP ratios for NNRTIs. Mean (SD) BM : MP ratios are illustrated for each drug. Where studies reported drug levels measured at different infant ages (representing different sampling times post-partum), these are represented as a separate line. The vertical line indicates a BM : MP ratio of 1, where BM and MP levels are equal. Pooled statistics are shown by the diamond and the *I*^2^ statistic is indicated. NVP, nevirapine; EFV, efavirenz; ETR, etravirine; NR, not reported; NaN, not a number; NA, not available. *Conference proceeding.

**Figure 3.**
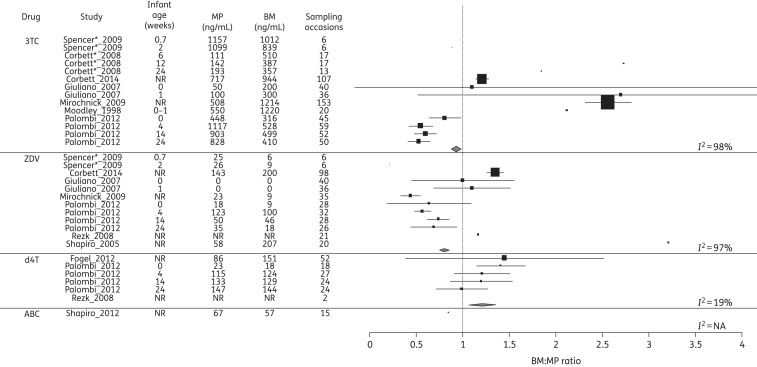
Forest plot of BM : MP ratios for NRTIs. Mean (SD) BM : MP ratios are illustrated for each drug. Where studies reported drug levels measured at different infant ages (representing different sampling times post-partum), these are represented as a separate line. The vertical line indicates a BM : MP ratio of 1, where BM and MP levels are equal. Pooled statistics are shown by the diamond and the *I*^2^ statistic is indicated. 3TC, lamivudine; ZDV, zidovudine; d4T, stavudine; ABC, abacavir; NR, not reported; NA, not available. *Conference proceeding.

**Figure 4.**
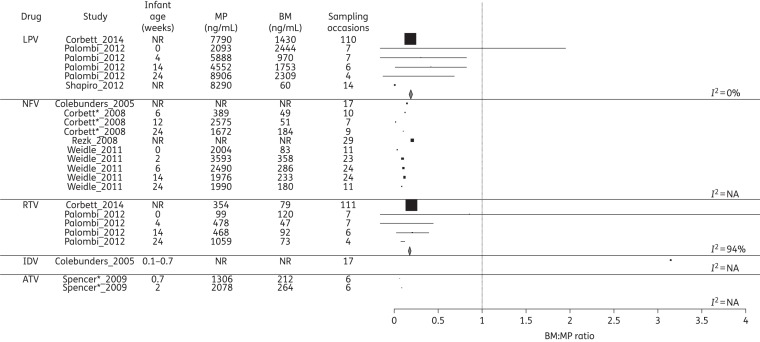
Forest plot of BM : MP ratios for PIs. Mean (SD) BM : MP ratios are illustrated for each drug. Where studies reported drug levels at different infant ages (representing different sampling times post-partum), these are represented as a separate line. The vertical line indicates a BM : MP ratio of 1, where BM and MP levels are equal. Pooled statistics are shown by the diamond and the *I*^2^ statistic is indicated. LPV, lopinavir; NFV, nelfinavir; RTV, ritonavir; IDV, indinavir; ATV, atazanavir; NR, not reported; NA, not available. *Conference proceeding.

**Figure 5.**
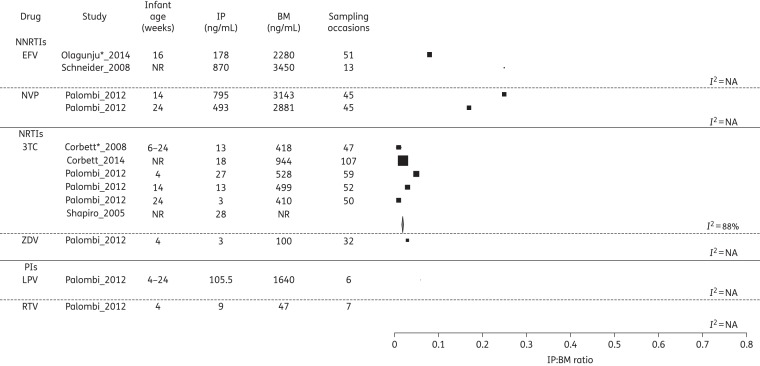
Forest plot of IP : BM ratios for all drugs where infant concentrations were detectable, grouped according to drug class. Where studies reported drug levels at different infant ages, these are represented as a separate line. Pooled statistics are shown by the diamond and the *I*^2^ statistic is indicated. EFV, efavirenz; NVP, nevirapine; 3TC, lamivudine; ZDV, zidovudine; LPV, lopinavir; RTV, ritonavir; NR, not reported; NA, not available. *Conference proceeding.

**Figure 6.**
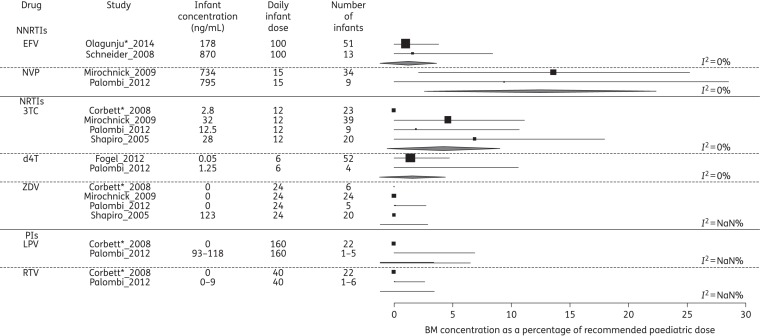
‘Dose’ via BM to a fully breast-fed 3 kg infant, as a percentage of recommended paediatric dose. Pooled statistics are shown by the diamond and the *I*^2^ statistic is indicated. EFV, efavirenz; NVP, nevirapine; 3TC, lamivudine; d4T, stavudine; ZDV, zidovudine; LPV, lopinavir; RTV, ritonavir; NaN, not a number. *Conference proceeding.

The ARV concentrations in MP and BM and the corresponding BM : MP ratios for each class of drug are summarized in Figures 2–4. Data on infant ARV concentrations resulting from BM exposure are summarized in Figure 5. Figure 6 illustrates the percentage of recommended infant dose ingested by a fully breast-fed 3 kg infant; results for other weights were similar and are not presented here.

#### NNRTIs

Seventeen studies reported on NNRTI levels in MP and BM, of which 14 measured nevirapine, 2 measured efavirenz and 1 measured etravirine.

Despite considerable heterogeneity (*I*^2^ = 95%), studies evaluating nevirapine transfer into BM consistently showed a BM : MP ratio of <1, with an overall pooled estimate of 0.73 (95% CI 0.71–0.76). Furthermore, three studies reported nevirapine levels in MP and BM at several timepoints post-delivery, demonstrating the BM : MP ratios to be relatively constant from 6 to 24 weeks post-partum, although numbers sampled at each timepoint were small.^22–24^ Eleven reporting IP nevirapine concentrations were confounded by administration of nevirapine to the infant. However, both Mirochnick *et al*.^25^ and Palombi *et al*.^23^ performed longitudinal analyses on MP, BM and IP; given the half-life of 30 h for nevirapine in infants from a similar population,^26^ by 3 months we presumed that detectable infant levels reflected BM transfer of drug. Both studies reported similar IP concentrations of ∼12% of MP at 14 weeks and <5% by 24 weeks.^23,25^

Both studies of efavirenz reported BM levels lower than plasma levels. The ratio of IP to BM was 0.24 in one study^27^ and 0.08 in the other.^28^ Etravirine was found to accumulate in the breast, with the BM : MP ratio rising from 1.09 to 3.27 between days 5 and 14 post-partum. Infant etravirine levels were not measured.

### NRTIs

Fourteen studies reported NRTI levels in MP and BM, of which 10 measured lamivudine, 7 measured zidovudine, 3 measured stavudine, 2 measured tenofovir, 1 measured abacavir and 1 measured emtricitabine.

Seven of 10 studies showed lamivudine accumulation in the breast, with a median BM : MP ratio of >1, although the variability both within and between studies was notable (*I*^2^ = 98%). The remaining three studies^23,29,45^ found that lamivudine BM : MP ratios were <1. The overall pooled estimate was 0.93 (95% CI 0.89–0.98). The majority of studies^15,16,23–25^ demonstrated the BM : MP ratios were constant from delivery throughout post-partum. However, in studies that performed intensive PK sampling,^15,16^ there was evidence of differential PK within the MP and BM compartments; a slower elimination rate of lamivudine was observed in BM than in MP, which resulted in a gradual increase in the BM : MP ratio across the dosing interval. Breastfeeding infants also had measurable drug, with IP concentrations between 2% and 6% of MP.^22,23,30^ Infant lamivudine exposures and corresponding IP : BM ratios declined over the course of the post-partum period, in spite of a constant delivery of drug through BM, which potentially reflects maturation (or ontogeny) of the infant's metabolic clearance system. Two studies evaluated MP and BM levels of zidovudine and lamivudine at intervals up to 24 weeks post-partum,^22,23^ finding the BM : MP ratios of these drugs to remain constant over this time.

Regarding zidovudine, three studies reported lower concentrations in BM compared with MP,^15,23,25^ three described higher levels^16,30,31^ and the final study^24^ demonstrated wide interindividual variability in the BM : MP ratio. The overall pooled estimate was 0.80 (95% CI 0.76–0.85). Infant levels of zidovudine were determined in three studies.^16,23,25^ Zidovudine was measurable at the time of delivery (potentially a reflection of transplacental passage of drug), but was largely undetectable in IP after the neonatal period had passed.

Three studies report stavudine BM : MP ratios of >1^23,31,32^ with a pooled estimate of 1.21 (1.07–1.36), which is significantly >1; however, infant concentrations approached the lower limit of quantification with a median value of zero.^23,32^ Two studies have measured tenofovir in BM. In one, tenofovir was measurable in the BM of only 4 out of 25 women sampled and neither study reported corresponding MP or IP concentrations.^33,34^ A single study measured emtricitabine in BM, but not MP. Abacavir had a reported BM:MP ratio of 0.85 in a single study, but BM, MP and IP concentrations were not reported.^45^

### PIs

Eight studies reported on PI levels in BM. One study measured atazanavir, one indinavir, four lopinavir used in combination with low-dose ritonavir and four nelfinavir. In all cases, the low-dose ritonavir was used as a boosting agent for lopinavir. Overall penetration of these PIs into BM was low (<40%) relative to MP. Only indinavir was found to accumulate in BM and this study must be interpreted with caution since the samples were drawn from a single mother on five consecutive days.^18^ Of the five studies analysing infant concentrations of PIs, four reported levels below the limit of quantification. One study from Malawi was able to detect lopinavir and ritonavir in the plasma of breast-fed infants, at ∼2% of MP levels.^23^

## Discussion

Although increasing numbers of countries have adopted WHO option B+, meaning that all pregnant and breastfeeding women will receive lifelong ART,^2^ understanding of the PK of transfer between mother and infant remains incomplete. Target concentrations have not been defined for any ARV in BM and many studies utilized an *in vitro* IC_50_ or IC_95_ with correction for protein binding. These approaches are not standardized and do not incorporate the active intracellular metabolites of NRTIs.^35,36^ Thus, we chose not to incorporate comparison against IC_50_ in the formal undertaking of this systematic review.

The NRTIs have higher and more variable BM penetration than the NNRTIs or PIs. Lamivudine has the highest accumulation in the BM and reaches detectable levels in the infant. Initially assumed to have unlikely clinical significance^30^ (exposure ∼5% of IC_50_^37^), more recent analysis of infants who acquired HIV through breastfeeding in the Kisumu Breastfeeding Study (KiBS) (mothers received zidovudine, lamivudine and either nevirapine or nelfinavir) found more than three-quarters to have resistance to both nevirapine and lamivudine.^7^ Further, amongst mothers who initiated triple therapy post-partum on the basis of clinical need in the PEPI-Malawi trial, almost one-third of HIV-positive infants were found to have multiclass drug resistance.^8^ The accumulation of zidovudine in BM was also highly variable between studies, but in most cases infant levels were undetectable, possibly attributed to the drug's rapid elimination half-life (∼1 h). Tenofovir and emtricitabine are measurable in BM, although the relationships with maternal and IP concentrations remain poorly defined; furthermore, tenofovir is not recommended for use in children aged <2 years. These findings have clinical and programmatic significance. Zidovudine and lamivudine remain components of first-line PMTCT and ARV regimens in low-resource settings and tenofovir and emtricitabine are increasingly used with implementation of WHO 2013 guidelines.^38^

Despite being a first-line ARV in PMTCT,^38^ only two studies assessed efavirenz concentrations in BM. These showed infant levels approach the minimum levels considered necessary for effective treatment in adults, reaching 13%^27^ and 8%^39^ of MP concentrations. No data about effective efavirenz levels necessary for protection of neonates by PMTCT are published. In part, this is a consequence of efavirenz having been contraindicated in pregnancy until 2012 due to teratogenicity concerns, which have recently been refuted by meta-analysis of exposed pregnancies;^40^ additionally, the drug is not recommended for use in children aged <3 years or weighing <10 kg except under exceptional circumstances and ideally with pharmacogenomic testing.^13^ Even with the extension to FDA licensing for young infants, current guidelines state it should not be used in infants aged <3 months. The studies by Schneider *et al*.^27^ and Olagunju *et al*.^28^ indicate that the ‘dose’ received by the breast-fed infant may reach 3.5% of the paediatric dose. The consequences of this in the neonatal period and the influence of maternal and infant pharmacogenomics on the concentrations reached warrant further evaluation.

The clinical relevance of ARV concentrations in BM is not fully understood. Whilst high levels have been correlated with reductions in the BM viral load, reducing the amount of virus to which the infant is exposed,^24^ differential accumulation of agents used within a triple-therapy regimen risks exposure to monotherapy within the compartment of the breast, potentially selecting resistant HIV strains. Furthermore, transfer of low levels of individual drugs to a breast-fed HIV-positive infant is associated with high rates of drug resistance as demonstrated in secondary analyses of both KiBS^7^ and PEPI-Malawi.^8^ Future studies should consider not only the levels of each individual drug, but also the optimal combination to be used to maximize benefits and reduce risks.

The wide variability within and between studies may result from differences in sampling time relative to dose, differences in drug concentration assays and the statistical method used to report concentrations below the lower limit of quantification, in addition to biological differences between populations. However, it is noted that variability was particularly marked for the NRTIs. Intensive PK data suggest there is a possible lag in the elimination of lamivudine (and to a lesser extent zidovudine) from BM, as shown by a gradual increase in the BM : MP ratios over the course of the dosing interval. This may, in part, explain the extensive variation in NRTI BM : MP ratios across studies, as sparse samples are taken at different times relative to dosing.

The accumulation of PIs in BM is low. Protein binding is the strongest predictor of drug transfer in BM,^41^ which explains lower levels of PIs penetrating BM compared with the other classes of drug (∼30% protein binding for zidovudine and lamivudine compared with >90% for the PIs).^42^ Maternal covariates influencing the variability in BM elimination of ARVs are poorly defined, but may include clinical states affecting circulating plasma proteins, such as intercurrent illness and poor nutrition.

### Limitations

#### Statistical limitations

There are a number of limitations to the analysis. A normal distribution is assumed when converting medians and IQRs to means and standard deviations and comparison of pooled estimates must be made with caution. Significant heterogeneity was observed with CIs that frequently do not overlap (Figures 2–5), necessitating restriction of analyses to the generalized inverse variance method (use of a random-effects model^43^ is an alternative approach).

We have presented a pooled estimate for every drug, which has an associated measure of spread. However, in some cases this means we are presenting a pooled estimate for a single study, which some may consider as not being meaningful. This is related to the limited data available.

The analysis of infant drug concentrations as a percentage of the recommended paediatric dose (Figure 6) relied on several assumptions. The source publications did not state infant weight and quantifying the volume of milk intake is challenging—we used the standard assumption of 150 mL/kg/day BM intake. Furthermore, most BM concentrations reflected a single timepoint during the dosing interval whereas the majority of mothers were taking a twice-daily regimen; our calculations assume a stable concentration of drug in BM throughout the dosing interval. Finally, we used an unlicensed efavirenz dose, which remains contraindicated in infants aged <3 months.

#### Limitations of reported methods

Although validated methods are essential for accurate and reliable drug concentration data,^44^ published clinical PK studies of ARVs in breastfeeding mother/infant pairs rarely report detail. Descriptions of sample collection, fraction of milk analysed, extraction method, type of internal standard, stability, matrix effects, recovery, accuracy and precision are frequently lacking.

The complexity of BM, particularly relating to variable lipid and protein content, requires that extraction methods are carefully validated. Among the studies reported here, most authors did not specify the milk fraction analysed or the extraction method used. This information is essential. Whereas Fogel *et al*.^32^ reported no significant difference in stavudine levels between whole and skimmed milk, Shapiro *et al*.^45^ noted significantly different recoveries of drug between skimmed and whole BM and consequently elected to report whole milk concentrations.^30,45^ Physicochemical principles influence the partitioning of drug into aqueous or lipid fractions of milk, but both are ingested by the infant and therefore whole milk is more likely to reflect the true clinical situation.

The majority of BM PK studies included in this review followed the stringent eligibility criteria of the PMTCT trial within which they were nested. This selection process, rather than recruitment under operational conditions, may have excluded women with covariates influencing BM elimination of ARVs.

Studies report BM concentrations of only 13 of the currently available ARVs. At the point of licensing, new drugs will not have been assessed in pregnant and breastfeeding populations. Whereas in well-resourced settings formula feeding is an option for mothers on these drugs, validated methodology is essential to enable investigation of BM elimination of drugs intended for widespread use in low-resource populations where prolonged breastfeeding is the norm.

To yield precise, high-quality data in an efficient manner, the proposed study of a novel ARV in breastfeeding mother/infant pairs can be informed by the findings of this review as summarized in Table 2.

**Table 2.** Recommendations for future studies

Component of study design	Ideal characteristics
Clinical	
population	population in whom the drug is to be used
study design	specific PK hypothesis or aims to be stated
sampling schedule	optimal sampling schedule to be considered from known PK of drugs
sample size	determined to enable estimation of PK parameters with a high degree of precision; rational basis from previous data (if available)
BM sampling	description of consistent, reproducible sampling method
time after birth	several timepoints relative to delivery; ideally the same mother/infant pairs followed longitudinally
time post-dose	sufficient timepoints to allow construction of an AUC for MP, BM and IP
documentation of timing	time of drug intake and PK sampling recorded
sampling of different matrices	contemporaneous sampling of MP and BM; validation of infant sampling in relation to maternal dose and recent feed
Laboratory	
assay validation	standards to be made up in donated BM to exclude matrix effect; use of internal standard labelled with a stable isotope
milk fraction	whole milk or clear justification for use of skimmed milk fraction with validation
BM storage	assay validation to include stability assessment
detection	highly sensitive assays such as HPLC-MS/MS to enable detection of low levels of drug
reporting	reporting of assay sensitivity to aid interpretation of BLQ levels
analysis	clear statement of method used to interpret BLQ levels

BLQ, below the limit of quantification.

### Conclusions

This systematic review reveals that the NRTI and NNRTI classes of ARVs are eliminated in the BM of HIV-positive women and transferred to breastfeeding infants, which may explain the development of drug-resistant HIV in infants where maternal ARVs have failed to prevent transmission. Available data are drawn from a small number of diverse studies employing laboratory methodology, which is frequently incompletely validated. Adoption of recent WHO guidelines will dramatically increase the use of ARVs in women of childbearing age in low-resource settings. Evaluation of the PK of both existing and novel drugs in breastfeeding mother/infant pairs is a matter of priority in order that best practice and safety can be ensured as novel regimens are rolled out.

## Funding

C. J. W. held an Academy of Medical Sciences Starter Grant for Clinical Lecturers investigating ARV drugs in breastfeeding Ugandan mother/infant pairs. C. J. W. commenced a Wellcome Trust Clinical Postdoctoral Fellowship (104422/Z/14/Z) to investigate PK of ARVs in breastfeeding mother/infant pairs on 1 February 2015.

## Transparency declarations

None to declare.

### Author contributions

C. J. W.: concept for review, study design and protocol development, data collection, data analysis and interpretation, preparation of figures and manuscript writing.

P. G.: input into all areas listed for C. J. W., with particular input into protocol development and methods.

L. J. B.: statistical analysis of drug levels.

S. H. K.: discussion of all elements of study design, data collection and interpretation, particularly where this was challenging. Input into manuscript.

L. J. E.: protocol development, literature searches, data collection and analysis and manuscript writing.

## Supplementary data

The protocol and Tables S1 and S2 are available as Supplementary data at *JAC* Online (http://jac.oxfordjournals.org/).

Supplementary Data
