# Supplementary material for: Is infant exposure to antiretroviral drugs during breastfeeding quantitatively important? A systematic review and meta-analysis of pharmacokinetic studies
Source: J Antimicrob Chemother. 2015 Apr 8;70(7):1928–41. doi: 10.1093/jac/dkv080 (PMC4472329; doi:10.1093/jac/dkv080)
Supplement: Supplementary Data [file supp_70_7_1928__index.html]

Is infant exposure to antiretroviral drugs during breastfeeding quantitatively important? A systematic review and meta-analysis of pharmacokinetic studies — Supplementary Data 

# Is infant exposure to antiretroviral drugs during breastfeeding quantitatively important? A systematic review and meta-analysis of pharmacokinetic studies

## Supplementary Data

Supplementary Data

**Files in this Data Supplement:**

- Supplementary Data - Docx file
- Supplementary Tables - docx file
