# Supplementary material for: Is infant exposure to antiretroviral drugs during breastfeeding quantitatively important? A systematic review and meta-analysis of pharmacokinetic studies
Source: J Antimicrob Chemother. 2015 Apr 8;70(7):1928–41. doi: 10.1093/jac/dkv080 (PMC4472329; doi:10.1093/jac/dkv080)
Supplement: Supplementary Data [file supp_dkv080_dkv080supp.docx]

**A Systematic Review of Exposure to Anti-retrovirals through Breastfeeding**

Background

Antiretroviral drugs (ARVs) used in prevention mother to child transmission of HIV (PMTCT) regimens can reduce transmission rates from 14-54% among untreated mothers (WHO 2010) to as low as 1-5%, even where infants are breastfed (Bedri, Gudetta et al. 2008; Chasela, Hudgens et al. 2010; Shapiro, Hughes et al. 2010; de Vincenzi 2011; Siegfried, van der Merwe et al. 2011; Thomas, Masaba et al. 2011). Nevertheless an estimated 430 000 infants continue to be infected annually (World Health Organization. 2010).

Breastmilk (BM) exposure is an important route of HIV transmission (Mofenson 2010; UNAIDS 2010); in the absence of ARVs the proportion of vertical infections acquired thus is estimated at 40%. HIV positive mothers in well-resourced settings are counselled to avoid breastfeeding (2006); conversely, the WHO recommends exclusive breastfeeding in the developing world, where formula feeding is associated with high infant mortality (WHO 2010). Even in the context of effective highly active anti-retroviral therapy (HAART), with suppression of circulating HIV1 RNA to undetectable levels, the risk of infection remains; this may relate to continued replication of the virus within the distinct anatomical site of the breast (Gantt, Carlsson et al. 2010). Several recent studies have shown that infants of ARV recipients who acquire HIV via BM are significantly more likely to develop drug resistance mutations than those infected *in utero* (Fogel, Li et al. 2011; Zeh, Weidle et al. 2011), thus limiting potential therapeutic options for these children. Furthermore, prolonged exposure to ARVs through BM has been associated with risk of toxicity to the infant (Dryden-Peterson, Shapiro et al. 2011).

WHO consolidated guidelines of 2013 contain several key recommendations for low-resource settings, including changing to an efavirenz (EFV) backbone for first line therapy, raising the CD4 count for eligibility from 350 to 500, and following one of three optimised regimes for PMTCT (WHO 2013). As of January 2013, 11 countries had endorsed the B+ option (2013), whereby all HIV infected mothers are commenced on life-long ARV regardless of their disease stage (Ahmed, Kim et al. 2013). Consequently, increasing numbers of women will received ARVs during breastfeeding, and data are required to further optimise the management of this sub-population, to monitor for infant toxicities occurring due to drug exposure, and in the event that an infant becomes HIV infected, to screen for drug resistance. Indeed, several prominent authors argue that caution should be exercised as not all of the most recent recommendations are supported by evidence (Coutsoudis, Goga et al. 2013; Van de Perre, Tylleskar et al. 2013).

We aim in this review to assess, in breastfeeding mothers on ARVs, a variety of factors that could potentially accelerate drug resistant HIV strains in the infant developing.

**Objectives**

In breast feeding mothers on highly active antiretroviral therapy (HAART) for their own health, or ARVs for preventing maternal to child transmission:

- To summarise ARV levels in breast milk
- Consequent exposure to the infant

**Inclusion criteria**

Studies that report drug levels of any ARV in breast milk of women on ARVs (either for treatment or as part of preventing maternal to child transmission).

**Study design**

Pharmacokinetic studies nested within cohort studies and trials; any prospective study.

**Participants**

Any HIV positive mother who is taking or has recently taken ARVs and is currently breast feeding, including those where mixed infant feeding has been introduced.

**Outcomes**

ARV levels in human breast milk

Ratio of ARV in maternal plasma to breastmilk (M:P ratio)

ARV levels in infants

**Methods**

PubMed Central, SCOPUS and LactMed databases will be searched using the key words ‘antiretroviral’ and ‘breast’ and ‘milk’, and subsequently, by replacing the generic term ‘antiretroviral’ with the name of each individual agent in turn. No date or language restrictions will be applied. The proceedings of relevant conferences will be searched using the Conferences in Retroviruses and Opportunistic Infections (CROI) database. Citation lists from review articles and papers meeting inclusion criteria will be manually searched.

**Data Collection Process**

An excel spreadsheet will be constructed to enable abstraction of data. The following data will be collected:

**Descriptive Data**

**Author, Date**

**Study design:** nested within PMCTC trial (and if so, which study), observational study or case series

**Participants:** Country; how women were recruited; regimens (which drugs, and for how long); sample size, inclusion and exclusion criteria

**Infants:** Time of sample relative to birth; whether infant ARVs were administered

**Outcomes:** ARV levels: median and IQR for levels in the mother, in the milk, and in the infant.

**Clinical methods:** Rich or sparse sampling strategy; method of obtaining breastmilk; timing of sample relative to infant feed; timing of all samples relative to dose; sampling schedule (number of samples, time range encompassed).

**Laboratory methods:** Milk fraction analysed; laboratory method; sensitivity of assay

A summary of descriptive data will be tabulated

**Study Quality Assessment**

The quality of the studies will be examined in relation to the following parameters:

**Clinical conduct:**

Methods used to obtain BM samples are clear: Yes/ No

Clear sampling strategy: Yes/ No

Sampling of all three matrices: Yes/ No/ Only 2

Sampling in all three matrices at the same time post-dose Yes/ No/ Unclear

**Laboratory methods**

Methods described in full, including reagents: Yes/No

Separate milk validation report: Yes/ No. If cross-reference made to another paper, that paper shall be cited

Assay sensitivity reported: Yes/ No

Brief summary of the detection method used.

Internal standard stated: Yes/ No. If yes, detail regarding whether this is an analog or a stable isotope labelled internal standard will be given

The data on study quality will be tabulated

**Summary Measures and Statistical Analysis**

The analysis will consider the ratios between drug levels (medians and IQR) in the mother, breast milk and infant taken concurrently. Where the ratio is not specifically stated in the paper, it will be calculated from the values given for maternal blood and breast milk.

The results will be tabulated, and the maternal plasma to breastmilk (MP:BM) ratio plotted graphically. Medians and IQR will be illustrated.

Formal statistical techniques will not be applied to these data.

Infant levels will be considered in relation to the published IC_50_, the concentration of drug found to inhibit the replication of 50% of virus in cell culture, which is commonly referred to as an index of the efficacy of antiretrovirals.

**Findings**

Results will be tabulated

BM:MP ratios will be graphed

Discussion of the findings will consider the results for each class of drugs in turn.

(2006). "Achievements in public health. Reduction in perinatal transmission of HIV infection - United States, 1985-2005." MMWR Morb Mortal Wkly Rep **55**: 592-597.

(2013). "Impact of an innovative approach to prevent mother-to-child transmission of HIV--Malawi, July 2011-September 2012." MMWR Morb Mortal Wkly Rep **62**(8): 148-151.

Ahmed, S., M. H. Kim, et al. (2013). "Risks and benefits of lifelong antiretroviral treatment for pregnant and breastfeeding women: a review of the evidence for the Option B+ approach." Curr Opin HIV AIDS **8**(5): 474-489.

Bedri, A., B. Gudetta, et al. (2008). "Extended-dose nevirapine to 6 weeks of age for infants to prevent HIV transmission via breastfeeding in Ethiopia, India, and Uganda: an analysis of three randomised controlled trials." Lancet **372**(9635): 300-313.

Chasela, C. S., M. G. Hudgens, et al. (2010). "Maternal or infant antiretroviral drugs to reduce HIV-1 transmission." N Engl J Med **362**(24): 2271-2281.

Coutsoudis, A., A. Goga, et al. (2013). "Is Option B+ the best choice?" Lancet **381**(9863): 269-271.

de Vincenzi, I. (2011). "Triple antiretroviral compared with zidovudine and single-dose nevirapine prophylaxis during pregnancy and breastfeeding for prevention of mother-to-child transmission of HIV-1 (Kesho Bora study): a randomised controlled trial." Lancet Infect Dis **11**(3): 171-180.

Dryden-Peterson, S., R. L. Shapiro, et al. (2011). "Increased risk of severe infant anemia after exposure to maternal HAART, Botswana." J Acquir Immune Defic Syndr **56**(5): 428-436.

Fogel, J., Q. Li, et al. (2011). "Initiation of antiretroviral treatment in women after delivery can induce multiclass drug resistance in breastfeeding HIV-infected infants." Clin Infect Dis **52**(8): 1069-1076.

Gantt, S., J. Carlsson, et al. (2010). "Genetic analyses of HIV-1 env sequences demonstrate limited compartmentalization in breast milk and suggest viral replication within the breast that increases with mastitis." J Virol **84**(20): 10812-10819.

Mofenson, L. M. (2010). "Protecting the next generation--eliminating perinatal HIV-1 infection." N Engl J Med **362**(24): 2316-2318.

Shapiro, R. L., M. D. Hughes, et al. (2010). "Antiretroviral regimens in pregnancy and breast-feeding in Botswana." N Engl J Med **362**(24): 2282-2294.

Siegfried, N., L. van der Merwe, et al. (2011). "Antiretrovirals for reducing the risk of mother-to-child transmission of HIV infection." Cochrane Database Syst Rev(7): CD003510.

Thomas, T. K., R. Masaba, et al. (2011). "Triple-antiretroviral prophylaxis to prevent mother-to-child HIV transmission through breastfeeding--the Kisumu Breastfeeding Study, Kenya: a clinical trial." PLoS Med **8**(3): e1001015.

UNAIDS (2010). Report on the global HIV/AIDS epidemic. Geneva, UNAIDS.

Van de Perre, P., T. Tylleskar, et al. (2013). "How evidence based are public health policies for prevention of mother to child transmission of HIV?" BMJ **346**: f3763.

WHO (2010). Antiretroviral drugs for treating pregnant women and preventing HIV infections in infants. Recommendations for a public health approach. WHO. Geneva.

WHO (2010). Guidelines on HIV and infant feeding 2010.

WHO (2013). Consolidated guidelines on the use of antiretroviral drugs for treating and preventing HIV infection. Geneva, World Health Organisation.

World Health Organization. (2010). PMTCT strategic vision 2010-2015 : preventing mother-to-child transmission of HIV to reach the UNGASS and Millennium Development Goals : moving towards the elimination of paediatric HIV, December 2009. Geneva, World Health Organization.

Zeh, C., P. J. Weidle, et al. (2011). "HIV-1 drug resistance emergence among breastfeeding infants born to HIV-infected mothers during a single-arm trial of triple-antiretroviral prophylaxis for prevention of mother-to-child transmission: a secondary analysis." PLoS Med **8**(3): e1000430.
