# Supplementary material for: Is infant exposure to antiretroviral drugs during breastfeeding quantitatively important? A systematic review and meta-analysis of pharmacokinetic studies
Source: J Antimicrob Chemother. 2015 Apr 8;70(7):1928–41. doi: 10.1093/jac/dkv080 (PMC4472329; doi:10.1093/jac/dkv080)
Supplement: Supplementary Data [file supp_dkv080_dkv080supp_tables.docx]

| Study Ref  **Table S1.** Pharmacokinetic Study Design | BM samples/ woman (N) | BM Sampling Detail | Time of Sampling Post Birth | Time of Sampling Post Dose | Matrices | Simultaneous Sampling |
| --- | --- | --- | --- | --- | --- | --- |
| Aizire ([Aizire, McConnell et al. 2012](#_ENREF_4)) | 9 | Yes | 0, 1, 2, 4, 6, 10, 14 weeks and 4, 5, and 6 months | SD | MP, BM | Yes |
| Benaboud ([Benaboud, Pruvost et al. 2011](#_ENREF_8)) | 4 | No | 1, 2, 3 and 7 days | 10 min – 21h | MP, BM | Yes |
| Colebunders ([Colebunders, Hodossy et al. 2005](#_ENREF_11)) | 10 | Yes | 1, 2, 3, 4 and 5 days | Trough + 2 hrs | MP, BM | Yes |
| Corbett* ([Corbett, Martinson et al. 2008](#_ENREF_13)) | 3 | No | 6, 12 and 24 weeks | 12-18 hrs | MP, IP, BM | Yes |
| Corbett ([Corbett, Kayira et al. 2014](#_ENREF_14)) | 4 | Yes | 6, 12 and 24 weeks | 0, 2, 4 and 6h (truncated PK) | MP, IP, BM | Yes |
| Fogel ([Fogel, Taha et al. 2012](#_ENREF_18)) | 1 | No | Birth – 24 months | Not recorded | MP, IP, BM | Yes |
| Frank ([Frank, Harms et al. 2012](#_ENREF_20)) | 1- 3 | No | Birth – 2 weeks | 0-2 weeks | MP, IP, BM | No |
| Guiliano ([Giuliano, Guidotti et al. 2007](#_ENREF_21)) | 2 | Yes | 0-3 and 7 days | 0-99 hrs | MP, BM | Yes |
| Kunz ([Kunz, Frank et al. 2009](#_ENREF_24)) | 3 | Yes | 1-6 weeks | SD | MP, IP, BM | Yes |
| Mirochnick ([Mirochnick, Fenton et al. 1998](#_ENREF_26)) | 1-2 | No | Not stated | Not stated | MP,BM | Not clear |
| Mirochnick ([Mirochnick, Thomas et al. 2009](#_ENREF_30)) | 5 | No | 0, 2, 6, 14 and 24 weeks | 1-12h | MP, IB(DBS), BM | Yes |
| Mirochnick ([Mirochnick, Taha et al. 2014](#_ENREF_29)) | 1 | No | 0-6 days | Not stated | MP, IP, BM | No |
| Moodley ([Moodley, Moodley et al. 1998](#_ENREF_33)) | 7 | No | 0– 7 days | Not stated (pre-feed) | MP, IP, BM | No |
| Musoke ([Musoke, Guay et al. 1999](#_ENREF_34)) | 3 | No | 2-7 days | 0-168 h | MP, IP, BM | Yes |
| Olagunju([Olagunju, Siccardi et al. 2014](#_ENREF_35)) | 1 | No | Mean 112 days | 12-14h | MP, IP, BM | Yes |
| Palombi ([Palombi, Pirillo et al. 2012](#_ENREF_36)) | 4 | Yes | 0, 1, 3 and 6 months | Not stated | MP, IP, BM | Yes |
| Rezk ([Rezk, White et al. 2008](#_ENREF_37)) | 1 | No | Not stated | Not stated | MP, BM | Yes |
| Ruff* ([Ruff, Hamzeh et al. 1994](#_ENREF_39)) | 4 | No | Not stated | 1-6 hrs | MP,BM | Yes |
| Schneider ([Schneider, Peltier et al. 2008](#_ENREF_40)) | 1 | Yes | 6-25 weeks | 3-4 hrs | MP, IP, BM | Yes |
| Shapiro ([Shapiro, Ribaudo et al. 2012](#_ENREF_42)) | 1 | No | 1 month | 11-17 hrs | MP, IP, BM | Yes |
| Shapiro ([Shapiro, Holland et al. 2005](#_ENREF_41)) | 1 | Yes | 2 or 5 months | 1-8.5 hrs | MS, IS, BM | Yes |
| Spencer* ([Spencer, Neely et al. 2009](#_ENREF_44)) | 10 | Yes | 5-14 days | 0, 2, 5, 8 and 24 hours | MP, BM | Yes |
| Spencer([Spencer, Liu et al. 2014](#_ENREF_43)) | 10 | No | 5 and 14 days | 0, 2, 4, 8 and 24 hours | MP, BM | Yes |
| Weidle ([Weidle, Zeh et al. 2011](#_ENREF_47)) | 5 | No | 0, 2, 6, 14 and 24 weeks | 2-8 hrs | MP, IB(DBS), BM | Yes |

Abbreviations: MP maternal plasma, IP infant plasma, IB infant blood, DBS dried blood spot, BM breast milk, SD single dose

*Conference proceeding rather than full manuscript

| Author (*Abstract)  **Table S2.** Quality of Laboratory Methods | Methods detailed | Validated BM Assay | BM Fraction | Extraction | Detection method | Assay Sensitivity  Stated | Internal standard stated |
| --- | --- | --- | --- | --- | --- | --- | --- |
| Aizire 2012 | Yes | No | Skim | PP | LC-MS/MS | Yes | No |
| Benaboud 2011 | Yes | No | Not stated | LLE | LC-MS/MS | Yes | No |
| Colebunders 2005 | No | Adapted HPLC (no reference) | Whole, homogenised | Not stated | HPLC-UV | No | No |
| Corbett* 2008 | No | No | Not stated | Not stated | LC-MS/MS | No | No |
| Corbett 2014 | Yes | Yes([Rezk, White et al. 2008](#_ENREF_37)) | Whole | SPE | HPLC-MS/MS | No | Analog (Delaviradine) |
| Fogel 2012 | Yes | Yes | Whole and skim | PP | UPLC-MS/MS | Yes | SIL IS (Deuterated d4T) |
| Frank 2012 | No | Adapted LC-MS/MS ([Kunz, Frank et al. 2009](#_ENREF_24)) | Not stated | Not stated | LC-MS/MS | No | No |
| Guiliano 2007 | No | Adapted HPLC ([Zhou and Sommadossi 1997](#_ENREF_53), [van Heeswijk, Hoetelmans et al. 1998](#_ENREF_46)) | Whole | Not stated | HPLC-UV | Yes | No IS used |
| Kunz 2009 | Yes | Yes | Whole | Not stated | LC-MS/MS | Yes | No |
| Mirochnick 1998 | Yes | No | Not stated | SPE | HPLC-UV | No | Analog (BIRH-414) |
| Mirochnick 2009 | Yes | Yes, not detailed | Not stated | Not stated | HPLC-UV | Yes | No |
| Mirochnick 2014 | No | No | Not stated | PP | LC-MS/MS | Yes | Isotopic |
| Moodley 1998 | No | Adapted HPLC ([Harker, Evans et al. 1994](#_ENREF_22)) | Not stated | Not stated | HPLC-UV | Yes | No |
| Musoke 1998 | No | No | Whole | Not stated | HPLC-UV | No | No |
| Olagunju 2014 | No | Yes | Not stated | Not stated | LC-MS/MS | No | No |
| Palombi 2012 | Yes | Yes ([Rezk, White et al. 2008](#_ENREF_37)) | Whole | PP | LC-MS/MS | Yes | Analog IS (Delavirdine) |
| Rezk 2008 | Yes | Adapted LC-MS/MS ([Jung, Rezk et al. 2007](#_ENREF_23)) | Skim | SPE | LC-MS/MS | Yes | Analog IS (Cimetidine) |
| Ruff* 1994 | No | No | Not stated | Not stated | No detail | No | No |
| Schneider 2008 | Yes | Adapted GC-MS ([Lemmer, Schneider et al. 2005](#_ENREF_25)) | Skim | PP | GC-MS | No | Analog (Hexobarbital) |
| Shapiro 2012 | Yes | Yes, not detailed | Whole and skim | Not stated | HLPC-UV | Yes | No |
| Shapiro 2005 | Yes | Yes | Whole and skim | Not stated | HPLC-UV | Yes | No |
| Spencer* 2009 | No | No | Not stated | Not stated | LC-MS/MS | Yes | No |
| Spencer 2014 | No | No | Not stated | Not stated | LC-MS/MS | No | No |
| Weidle 2011 | Yes | Yes | Not stated | PP | LC-MS/MS | Yes | SIL (Deuterated NFV) |

Abbreviations: HPLC high performance liquid chromatography, LC-MS/MS liquid chromatography with tandem mass spectrometry detection, GC-MS gas chromatography with mas spectrometry detection, HPLC-UV high performance liquid chromatography with ultraviolet detection, PP protein precipitation, LLE liquid liquid extraction, SPE solid phase extraction, SIL stable isotope labelled, NFV nelfinavir
